# Type 2 diabetes mellitus is associated with distinct post-treatment metabolic profiles in pulmonary tuberculosis

**DOI:** 10.3389/fcimb.2026.1887961

**Published:** 2026-08-11

**Authors:** Xue Li, Zuokuan He, Qin Gao, Zhili Hou, Kairu Zhang, Zhonghua Qin, Xingjie Zheng, Hongzhi Yu, Junping Wu, Huaiyong Chen

**Affiliations:** 1 Tianjin Key Laboratory of Lung Regenerative Medicine, Haihe Hospital, Tianjin University, Tianjin, China; 2 Department of Basic Medicine, Haihe Clinical College, Tianjin Medical University, Tianjin, China; 3 Department of Tuberculosis, Haihe Hospital, Tianjin University, Tianjin, China; 4 Center for Accurate Detection of Tuberculosis, Haihe Hospital, Tianjin University, Tianjin, China; 5 Key Research Laboratory for Infectious Disease Prevention for State Administration of Traditional Chinese Medicine, Tianjin Institute of Respiratory Diseases, Tianjin, China

**Keywords:** lipid metabolism, metabolic reprogramming, metabolomics, tuberculosis, type 2 diabetes mellitus

## Abstract

**Background:**

Type 2 diabetes mellitus (T2DM) is a major risk factor for pulmonary tuberculosis (PTB), exacerbates disease severity and reduces the efficacy of anti-tuberculosis therapy. Previous studies have shown that T2DM is associated with exacerbated metabolic disturbances in patients with PTB. However, metabolic differences between PTB patients with comorbid T2DM (PTB-DM) and those with PTB after anti-TB therapy remain unclear.

**Methods:**

Untargeted metabolomic analysis was conducted on plasma samples collected from April 2024 to November 2025 at Tianjin Haihe Hospital, China. A total of 75 participants were enrolled, including healthy controls, patients with newly diagnosed drug-sensitive PTB, patients with PTB-DM, as well as two independent post-treatment groups sampled after 6 months of standard anti-TB therapy: patients with PTB and patients with PTB-DM.

**Results:**

Patients with PTB exhibited marked metabolic disturbances, particularly in tyrosine metabolism and steroid hormone biosynthesis. Patients with PTB-DM showed broader metabolic reprogramming, including changes in arginine and proline metabolism, steroid hormone biosynthesis, cAMP signaling, and taurine and hypotaurine metabolism. After 6 months of standard therapy, the PTB_6M group showed treatment-associated metabolic differences, especially in bile acid metabolism, suggesting partial normalization of several metabolic features. In contrast, patients with PTB-DM after treatment showed persistent metabolic abnormalities involving sphingolipid, purine and biotin metabolism. Notably, 12-HETE and 12-HHTrE showed elevated levels in active PTB, decreased levels after treatment, but remained relatively higher in PTB-DM after treatment.

**Conclusions:**

This study offers an integrated assessment of metabolic alterations in PTB and PTB-DM, suggesting that comorbid T2DM is associated with broader metabolic dysregulation during active PTB and persistent post-treatment metabolic abnormalities.

## 1 Introduction

Tuberculosis (TB), caused by *Mycobacterium tuberculosis* (Mtb), remains a leading global public health challenge (Miggiano et al., 2020). Pulmonary tuberculosis (PTB) is the most common clinical manifestation of TB (Loddenkemper et al., 2015). There are approximately 10.87 million new TB cases worldwide, with an estimated 1.23 million deaths, making TB the leading cause of mortality from a single infectious agent.

In recent years, type 2 diabetes mellitus (T2DM) has emerged as a major risk factor for the development of PTB and poor prognosis. Individuals with T2DM have more than a threefold increased risk of developing active PTB, and PTB patients with comorbid T2DM (PTB-DM) exhibit higher bacillary loads, delayed sputum conversion, and elevated recurrence and mortality rates (Jeon and Murray, 2008; Viswanathan et al., 2014; Wang et al., 2024; Workneh et al., 2016). Metabolomic studies have revealed distinct metabolic signatures in patients with PTB-DM (Vinhaes et al., 2024). Patients with PTB-DM show pronounced disturbances in bile acid and arachidonic acid metabolism, accompanied by significant reductions in lysophosphatidylcholine (LPC) and phosphatidylcholine (PC) levels (Yen et al., 2023). Moreover, the interplay between PTB and T2DM involves immune–inflammatory signaling pathways, such as interleukin-17, PPAR, and PI3K-AKT pathways, indicating a complex immune–metabolic–endocrine imbalance underlying PTB-DM pathophysiology (Wang et al., 2023). This relationship may be bidirectional. Hyperglycaemia, insulin resistance, and chronic systemic inflammation can impair macrophage function and host antimicrobial responses to Mtb (Thong et al., 2025). Conversely, systemic inflammation and physiological stress responses associated with active PTB may contribute to glycaemic dysregulation, particularly in individuals with pre-existing insulin resistance or other underlying metabolic susceptibility (Liu et al., 2021).

Regarding metabolomic alterations after anti-tuberculosis therapy in patients with PTB-DM, Vrieling et al. reported persistently low plasma choline levels even after therapy, suggesting sustained metabolic disturbance (Vrieling et al., 2019). Similarly, Brake et al. integrated proteomic and lipidomic analyses and found that, although 2 months of anti-tuberculosis therapy partially reduced systemic inflammation, abnormalities in lipid metabolism persisted (Brake et al., 2025). Nevertheless, previous studies have mainly focused on metabolic abnormalities during active PTB and PTB-DM, whereas post-treatment metabolic profiles remain less well characterized. In particularly, it remains unclear whether patients with PTB-DM exhibit distinct and persistent systemic metabolic abnormalities after completing standard anti-tuberculosis therapy compared with patients with PTB alone.

To address this knowledge gap, we performed untargeted plasma metabolomic profiling in healthy controls, newly diagnosed patients with PTB, newly diagnosed patients with PTB-DM, and independent groups of patients with PTB or PTB-DM who had completed 6 months of anti-tuberculosis therapy. We aimed to characterize metabolic alterations associated with active PTB, identify additional perturbations associated with comorbid T2DM, and determine whether patients with PTB-DM exhibit persistent post-treatment metabolic abnormalities compared with patients with PTB alone.

## 2 Methods

### 2.1 Participants and samples

Participants were enrolled from April 2024 to November 2025 at Tianjin Haihe Hospital, China. Plasma samples were obtained from five groups: healthy controls (Healthy group), newly diagnosed patients with drug-sensitive PTB (PTB group), newly diagnosed patients with drug-sensitive PTB-DM (PTB-DM group), patients with drug-sensitive PTB who had completed 6 months of standard anti-tuberculosis therapy (PTB_6M group), and patients with drug-sensitive PTB-DM who had completed 6 months of standard anti-tuberculosis therapy (PTB-DM_6M group). This was an observational study. The newly diagnosed and 6 months treatment groups did not represent paired samples collected from the same individuals. We matched the age and sex of all groups being compared to ensure there were no statistically significant differences between them. All PTB diagnoses were established according to the World Health Organization diagnostic criteria, and drug susceptibility testing confirmed the sensitivity to all first-line anti-TB drugs. Participants with PTB-DM had a documented history of T2DM before PTB diagnosis and had received glucose-lowering treatment. Participants were excluded if they had drug-resistant tuberculosis, HIV infection, non-tuberculous mycobacterial disease, other active infectious diseases, malignant tumors, autoimmune disorders, severe hepatic or renal dysfunction, pregnancy or lactation, or current systemic glucocorticoid or immunosuppressive therapy. Patients with other major endocrine or metabolic disorders, except for T2DM in the PTB-DM groups, were also excluded. Fasting venous blood samples (5 mL) were collected from each participant early in the morning. Plasma was obtained by centrifugation at 2,000 rpm for 10 min at 4 °C, aliquoted, and stored at −80 °C for metabolomic analysis. The study was performed in accordance with the Declaration of Helsinki and approved by the Institutional Review Board of Tianjin Haihe Hospital (2025HHSQKT-023). Written informed consent was obtained from all participants.

### 2.2 Metabolites extraction and UHPLC–MS/MS analysis

Plasma metabolite extraction and UHPLC–MS/MS analysis were performed using a standardized untargeted metabolomics workflow implemented by Novogene Co., Ltd. The workflow was adapted from previously published plasma metabolomics protocols (Li et al., 2023; Yan et al., 2022), with minor modifications in the extraction-solvent ratio, methanol concentration, and centrifugation conditions. Plasma samples (100 μL) were mixed with 400 μL of 80% methanol in Eppendorf tubes, vortexed, chilled on ice for 5 min, and then centrifuged at 15,000 × g for 20 min at 4 °C. An aliquot of the supernatant was diluted with liquid chromatography–mass spectrometry (LC–MS)-grade water to a final methanol concentration of 53% and centrifuged again under the same conditions. The clarified supernatant was subsequently injected into the UHPLC–MS/MS platform. Untargeted metabolomic profiling was carried out by Novogene Co., Ltd. (Beijing, China) using a Vanquish UHPLC system coupled with an Orbitrap Q-Exactive HF-X mass spectrometer (Thermo Fisher Scientific, Germany). Chromatographic separation was performed on a Hypersil Gold C18 column (100 × 2.1 mm2, 1.9 μm) at a flow rate of 0.2 mL/min with a 17-min linear gradient. Mass spectrometry parameters were operated with the following settings: spray voltage 3.5 kV, sheath gas 35 psi, auxiliary gas 10 L/min, capillary temperature 320 °C, S-lens RF level 60, and auxiliary gas heater temperature 350 °C. Data were collected in both positive and negative electrospray ionization modes over an m/z range of 100–1500 using data-dependent MS/MS acquisition.

### 2.3 Quality control of metabolomics analysis

All study samples were analyzed in a single analytical batch. As described previously, equal volumes of plasma from each sample were pooled to generate a pooled sample, which was used as a quality control (QC) sample for metabolomic analysis. This approach was applied to ensure the stability of the system, monitor instrument performance, and evaluate analytical reliability throughout the experiment. In addition, blank samples were included to identify and remove background ions. The QC samples were divided into multiple aliquots and analyzed periodically before, during, and after LC-MS/MS acquisition of the study samples. Metabolites exhibiting a coefficient of variation (CV) exceeding 30% in QC samples were removed from further analysis.

### 2.4 Metabolites identification and quantification

Peak extraction, alignment, and quantification were performed based on the retention time and m/z. Metabolites were annotated using a 10-ppm mass tolerance and by matching their MS/MS spectra to the NovoMetDB library. Background ions relative to the blank samples were removed. Quantitative data were normalized to the QC samples, and the relative peak areas were calculated for subsequent statistical analyses.

### 2.5 Differential metabolite and pathway analysis

Metabolite annotation was performed using the Kyoto Encyclopedia of Genes and Genomes (KEGG), the Human Metabolome Database (HMDB), and the LIPID MAPS database. Multivariate analysis and principal component analysis (PCA) were conducted to derive variable importance in projection (VIP) scores for each metabolite. Univariate comparisons between groups were performed using Student’s t-test to calculate p-values and fold changes (FC). Candidate differential metabolites were identified using the predefined combined criteria of VIP > 1.0, p < 0.05, and FC > 1.5. To assess the robustness of the metabolite-level associations after multiple testing, nominal p values were additionally adjusted using the Benjamini–Hochberg false discovery rate procedure. Both nominal and FDR-adjusted p values are reported in the **Supplementary Tables**. The data were normalized using Z-scores before visualization. KEGG pathway enrichment analysis was performed on the identified DEMs to explore biological processes. Representative metabolites displayed in the figures were selected from the major enriched pathways identified in each group comparison. Selection was based on pathway relevance, relevance to the central biological question of the comparison, clarity of metabolite annotation, and support from published literature. These metabolites were presented to illustrate the principal pathway-level alterations and were not necessarily those with the smallest p values or largest fold changes. Complete lists of differential metabolites and their statistical information are provided in the **Supplementary Tables**. Volcano plots, heat maps, and bubble charts were generated in R using the ggplot2 package, whereas boxplots were produced using GraphPad Prism 9.

### 2.6 Statistical analysis

The data distribution normality was assessed using the Shapiro–Wilk test. Variables following a normal distribution are presented as mean ± (standard deviation, SD), whereas non-normally distributed variables are presented as median (Q1, Q3). Categorical variables are expressed as frequency (%). For comparisons between two groups, Student’s t-test or the appropriate non-parametric test was applied, whereas comparisons among multiple groups were performed using one-way analysis of variance (ANOVA) or the Kruskal–Wallis test followed by Dunn’s *post hoc* test. These statistical approaches were applied to both clinical characteristics and metabolomic data analyses. A p-value < 0.05 was considered statistically significant.

## 3 Results

### 3.1 Demographic characteristics of the participants

Plasma samples from 75 individuals were subjected to untargeted metabolomic profiling. Participants were classified into five independent groups: healthy controls (Healthy group), newly diagnosed drug-sensitive PTB patients (PTB group), PTB patients after 6 months of standard anti-TB therapy (PTB_6M group), newly diagnosed drug-sensitive PTB patients with comorbid T2DM (PTB-DM group), and PTB-DM patients after 6 months of anti-TB therapy (PTB-DM_6M group). As described in the Methods, the PTB_6M and PTB-DM_6M groups were independent post-treatment subgroups rather than paired follow-up samples from the PTB and PTB-DM groups. The baseline demographic characteristics are summarized in **Table 1**, and detailed clinical and laboratory data for the study participants are provided in **Supplementary Table 1**-**S4**. The mean age of patients in the PTB group was 48.6 ± 20.7 years, including 10 men (62.5%) and 6 women (37.5%). In the PTB_6M group, the mean age was 49.1± 21.0 years, with 7 men (58.3%) and 5 women (41.7%). Patients in the PTB-DM group had a mean age of 60.0 ± 13.1 years, comprising 13 men (86.7%) and 2 women (13.3%), whereas those in the PTB-DM_6M group had a mean age of 61.3 ± 11.0 years, including 8 men (66.7%) and 4 women (33.3%). The healthy control group had a mean age of 41.6 ± 6.9 years, with 6 men (30.0%) and 14 women (70.0%).

**Table 1:** Demographic characteristics of the study participants.

Characteristic	Healthy control (n=20)	PTB (n=28)		PTB-DM (n=27)	
		PTB (n=16)	PTB_6M (n=12)	PTB-DM (n=15)	PTB-DM_6M (n=12)
Sex, n (%)					
Male	6 (30.0)	10 (62.5)	7 (58.3)	13 (86.7)	8 (66.7)
Female	14 (70.0)	6 (37.5)	5 (41.7)	2 (13.3)	4 (33.3)
Age, y					
Mean ± SD	41.6 ± 6.9	48.6 ± 20.7	49.1 ± 21.0	60.0 ± 13.1	61.3 ± 11.0
Range	35.0–57.0	18.0–84.0	23.0–76.0	31.0–78.0	43.0–79.0

PTB, pulmonary tuberculosis; DM, diabetes mellitus; SD, standard deviation.

### 3.2 Global plasma metabolomic patterns across disease and treatment groups

**Figure 1:**
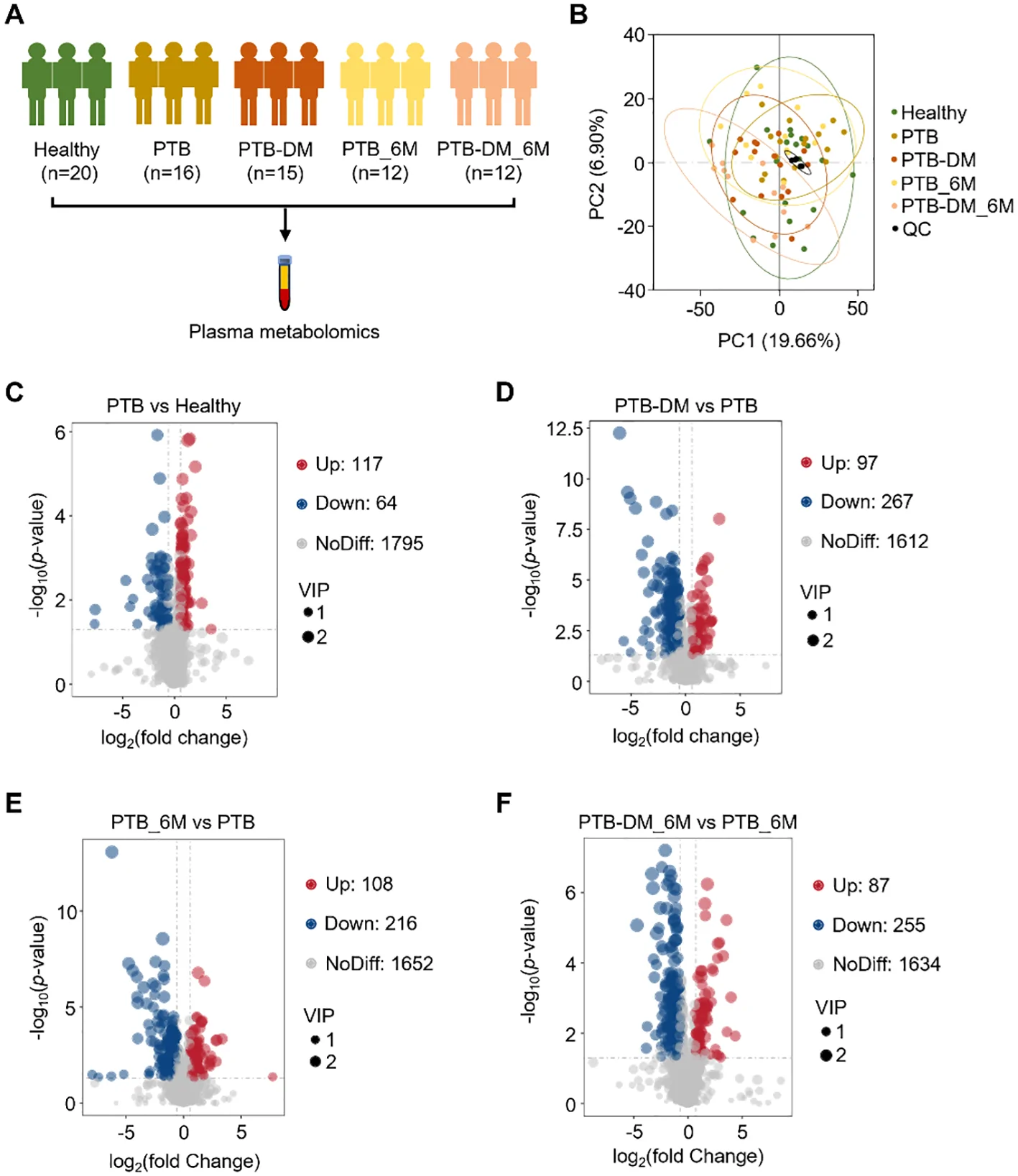
Overview of the study design. **(A)** Schematic diagram illustrating the study design. **(B)** Principal component analysis (PCA) plot of plasma metabolomic data. Each dot represents an individual sample, and colors denote different groups. **(C–F)** Volcano plots showing differentially expressed metabolites between the indicated groups. Red dots represent upregulated metabolites, blue dots represent downregulated metabolites, and grey dots indicate non-significant metabolites.

To comprehensively evaluate metabolic alterations across different disease states, untargeted plasma metabolomic analysis was performed for all five groups (**Figure 1A**). In total, 1,976 metabolites were identified based on integrated compound libraries. PCA revealed a clear separation trend among the groups (**Figure 1B**), indicating distinct metabolic phenotypes associated with disease progression and treatment status. Compared with the Healthy group, 181 DEMs were identified in the PTB group, including 117 upregulated and 64 downregulated metabolites (**Figure 1C**; **Supplementary Table 5**). Compared with the PTB group, 364 DEMs were identified in the PTB-DM group, comprising 97 upregulated and 267 downregulated metabolites (**Figure 1D**; **Supplementary Table 6**). After 6 months of standard anti-TB therapy, the PTB_6M group exhibited 324 DEMs relative to the baseline metabolite levels of the PTB group (108 upregulated and 216 downregulated) (**Figure 1E**; **Supplementary Table 7**). Similarly, comparison between the PTB-DM_6M and PTB_6M groups revealed 342 DEMs, with 87 upregulated and 255 downregulated metabolites in the PTB-DM_6M group (**Figure 1F**; **Supplementary Table 8**).

### 3.3 Metabolic disturbances associated with active PTB

**Figure 2:**
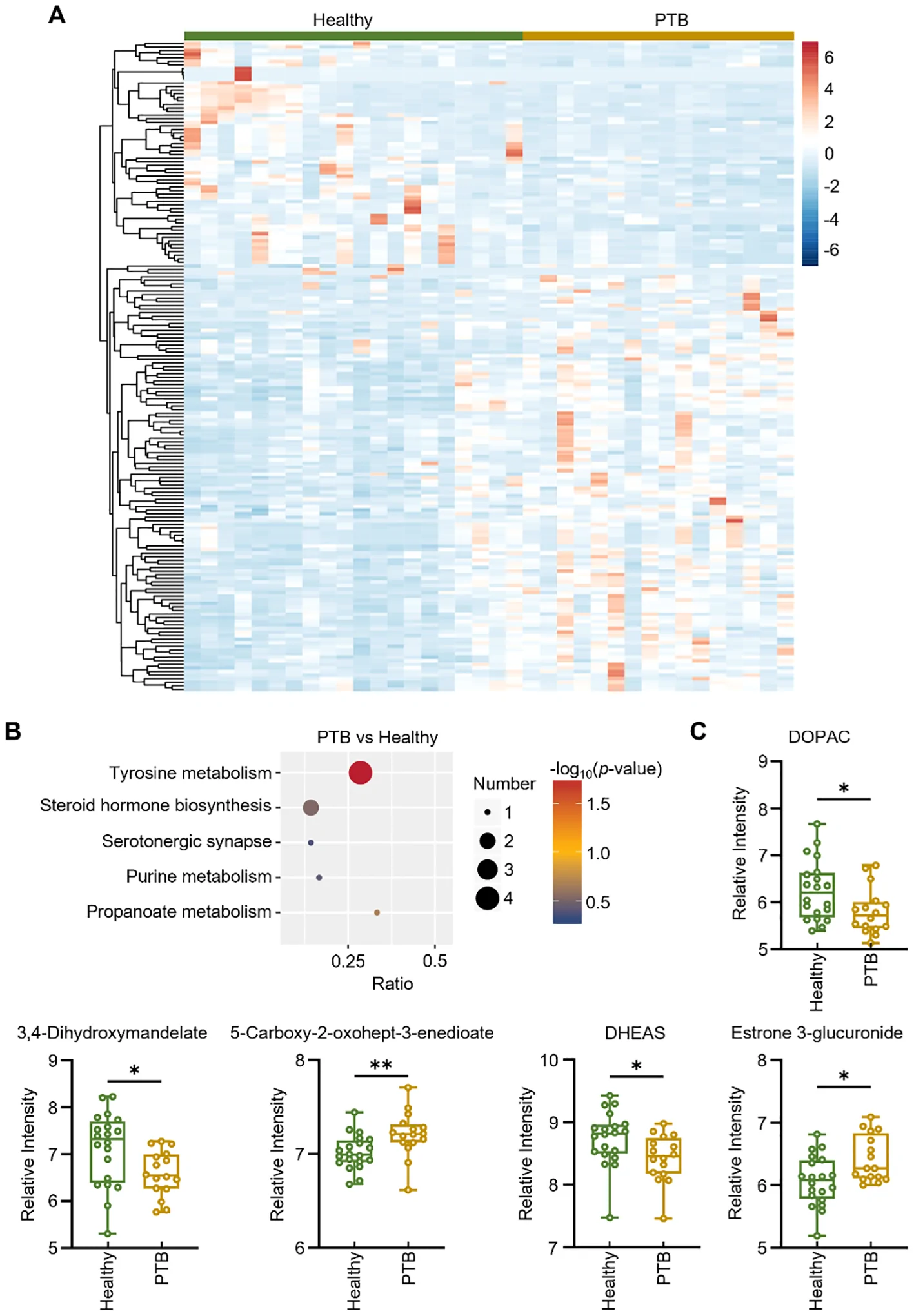
Metabolic disturbances associated with active PTB. **(A)** Heatmap showing the distribution of 181 differentially expressed metabolites between patients with PTB and healthy controls. Color intensity from blue to red indicates low to high relative abundance. **(B)** Bubble plot displaying the top five KEGG pathways significantly enriched among differentially expressed metabolites between the PTB and Healthy groups. Bubble size indicates the number of enriched metabolites, and color represents the −log_10_ p-value. **(C)** Relative abundance of representative differentially expressed metabolites from different enriched pathways. Data are presented as mean ± SD; *p < 0.05, **p < 0.01. PTB, pulmonary tuberculosis; KEGG, Kyoto Encyclopedia of Genes and Genomes; SD, standard deviation.

We first characterized the metabolic features of active PTB. Compared with healthy controls, patients with PTB exhibited a distinct plasma metabolic profile, with the most prominent pathway-level alterations involving tyrosine metabolism and steroid hormone biosynthesis. Heatmap visualization revealed a clear clustering of these metabolites across samples and groups (**Figure 2A**), indicating distinct metabolic patterns between patients with PTB and healthy individuals. The DEMs identified were primarily lipids, lipid-like molecules, organic acids and their derivatives, and heterocyclic compounds. KEGG pathway enrichment analysis indicated that these DEMs were mainly involved in tyrosine metabolism, steroid hormone biosynthesis (**Figure 2B**; **Supplementary Table 9**). Within the tyrosine metabolism pathway, the levels of 3,4-dihydroxybenzeneacetic acid (DOPAC) and 3,4-dihydroxymandelate (3,4-DHM) were significantly decreased in patients with PTB, whereas the 5-carboxy-2-oxohept-3-enedioate level was markedly elevated (**Figure 2C**). In the steroid hormone biosynthesis pathway, dehydroepiandrosterone sulfate (DHEAS) was significantly downregulated, whereas estrone 3-glucuronide was upregulated (**Figure 2C**). Collectively, these findings indicate that active PTB is characterized by dysregulation of tyrosine metabolism and steroid hormone biosynthesis.

### 3.4 Additional metabolic perturbations associated with concomitant T2DM

**Figure 3:**
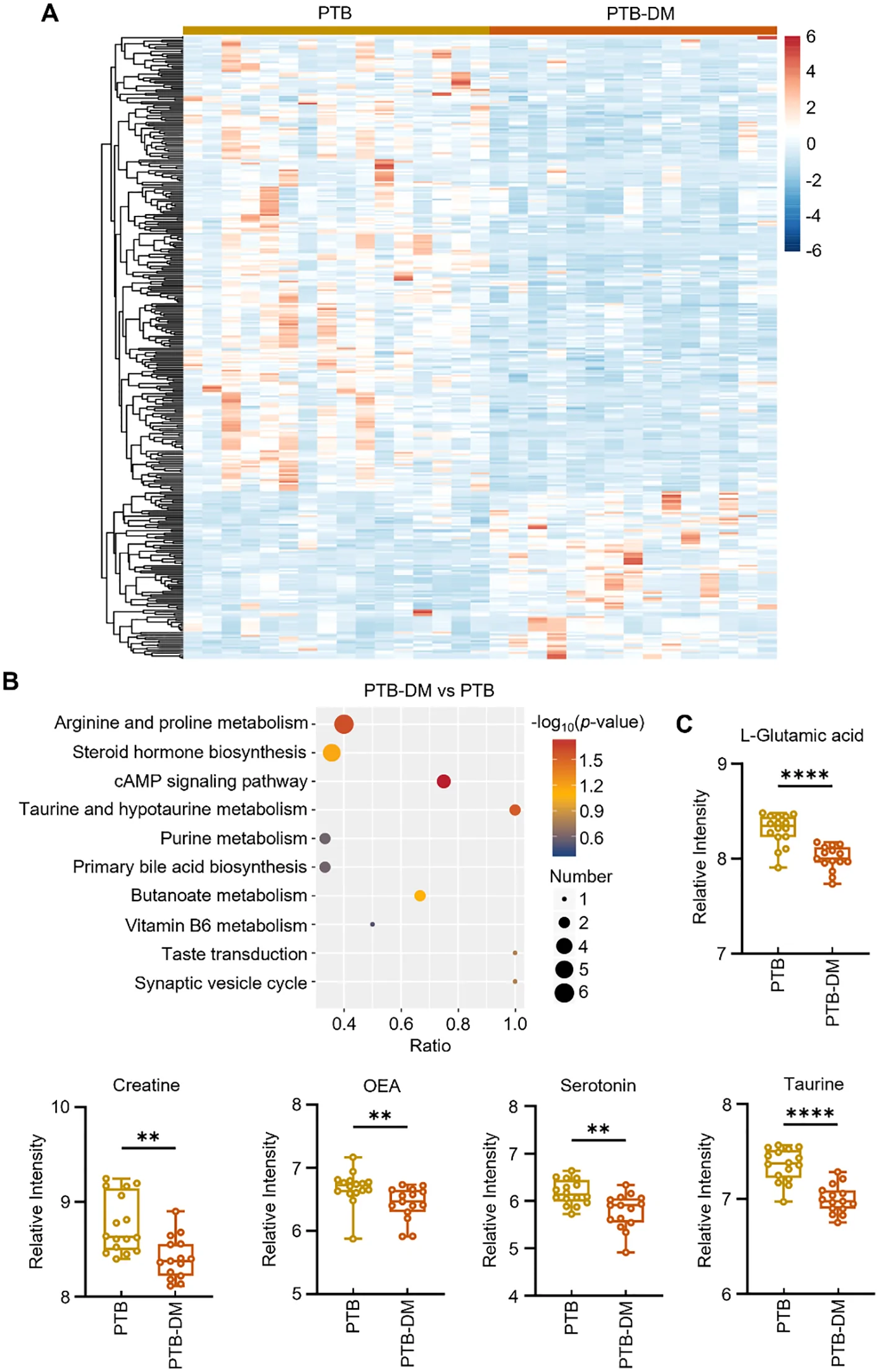
Additional metabolic perturbations associated with concomitant T2DM. **(A)** Heatmap displaying the relative abundance of differential metabolites between the PTB-DM and PTB groups. **(B)** Bubble plot displaying the top 10 KEGG pathways significantly enriched for differentially expressed metabolites between the PTB-DM and PTB groups. Bubble size indicates the number of enriched metabolites, and color represents the −log_10_ p-value. **(C)** Relative abundance of representative differentially expressed metabolites from different enriched pathways. Data are shown as mean ± SD; **p < 0.01, ****p < 0.0001. PTB, pulmonary tuberculosis; DM, diabetes mellitus; KEGG, Kyoto Encyclopedia of Genes and Genomes; SD, standard deviation.

We next investigated whether comorbid T2DM was associated with additional metabolic alterations beyond those observed in active PTB. Plasma metabolomic profiles were compared between patients with PTB-DM and PTB. A total of 364 DEMs were identified (**Figure 3A**), including lipids, lipid-like molecules, organic acids and their derivatives, heterocyclic compounds, and aromatic molecules. KEGG enrichment analysis revealed that these DEMs were primarily involved in arginine and proline metabolism, steroid hormone biosynthesis, cAMP signaling, and taurine and hypo taurine metabolism (**Figure 3B**; **Supplementary Table 10**). The levels of these metabolites, including L-glutamic acid, creatine, oleoylethanolamide (OEA), serotonin, and taurine were significantly reduced in patients with PTB-DM (**Figure 3C**). Taken together, these findings indicate that metabolic alterations associated with comorbid T2DM in PTB patients are mainly characterized by changes in arginine and proline metabolism, steroid hormone biosynthesis, cAMP signaling, taurine and hypotaurine metabolism. L-glutamic acid, creatine, oleoylethanolamide (OEA), serotonin, and taurine represent representative metabolites associated with these metabolic alterations and may warrant further investigation.

### 3.5 Treatment-associated metabolic changes in PTB

**Figure 4:**
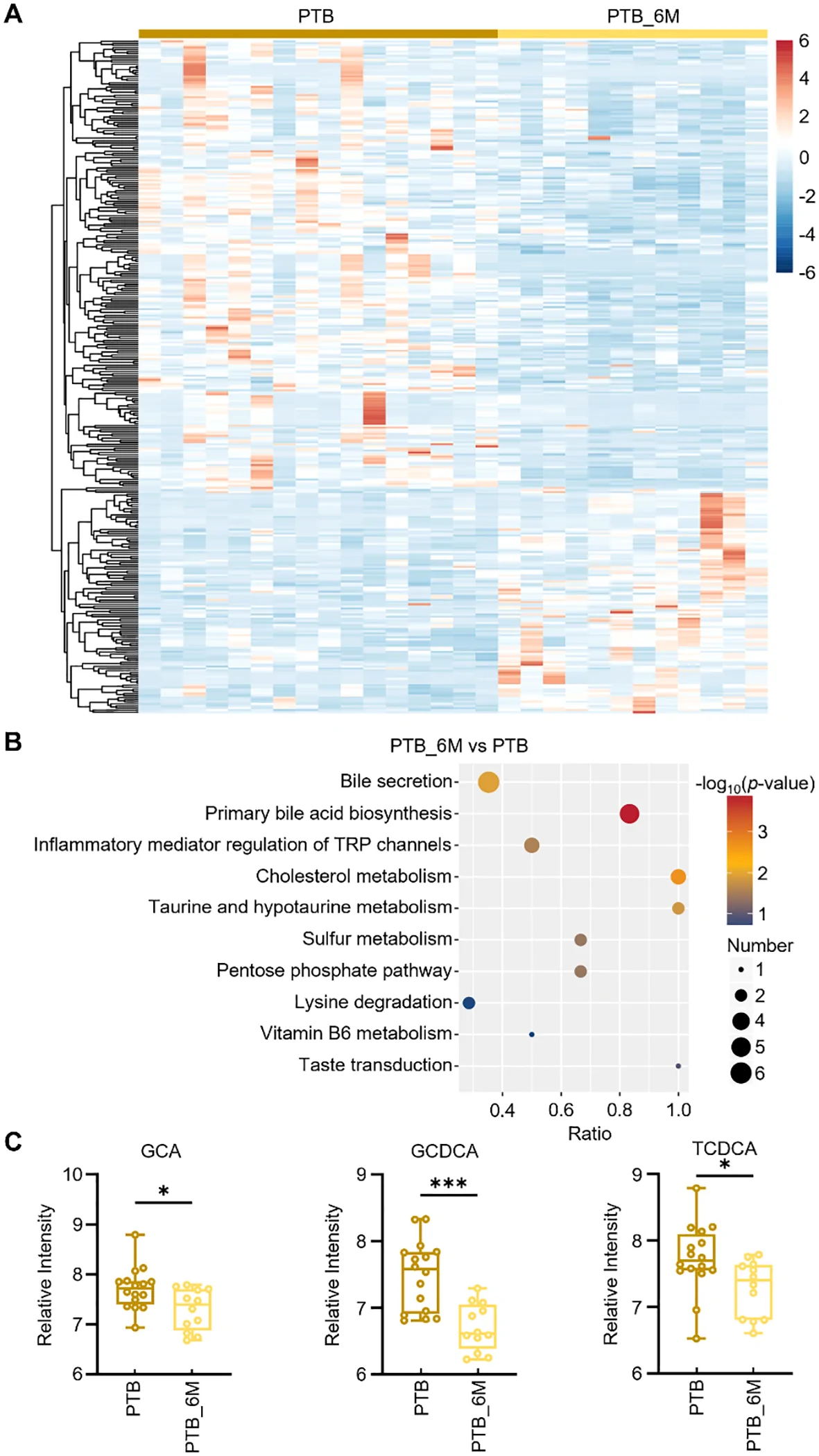
Treatment-associated metabolic changes in PTB. **(A)** Heatmap showing the relative abundance of differentially expressed metabolites between the PTB and PTB_6M groups. **(B)** Bubble plot presenting the top 10 KEGG significantly pathways enriched among differentially expressed metabolites between the PTB and PTB_6M groups. **(C)** Relative abundance of representative differentially expressed metabolites from different enriched pathways. Data are expressed as mean ± SD; *p < 0.05, ***p < 0.001. PTB, pulmonary tuberculosis; KEGG, Kyoto Encyclopedia of Genes and Genomes; SD, standard deviation.

We next examined post-treatment metabolic differences associated with PTB by comparing the PTB and PTB_6M groups. These DEMs were predominantly lipids, lipid-like molecules, organic acids and their derivatives, organic heterocyclic compounds, and related categories (**Figure 4A**). In patients with PTB alone, the most prominent post-treatment metabolic differences involved bile acid secretion, primary bile acid biosynthesis (**Figure 4B**; **Supplementary Table 11**). Representative bile acid metabolites—including glycocholic acid (GCA), glycochenodeoxycholic acid (GCDCA), and Tauro chenodeoxycholic acid (TCDCA)—were significantly lower in the PTB_6M group compared with the PTB group (**Figure 4C**). Collectively, these findings indicate that bile acid metabolism represents a major metabolic feature distinguishing PTB and PTB_6M groups, with GCA, GCDCA, and TCDCA showing significant differences after treatment.

### 3.6 Persistent post-treatment metabolic abnormalities associated with comorbid T2DM

**Figure 5:**
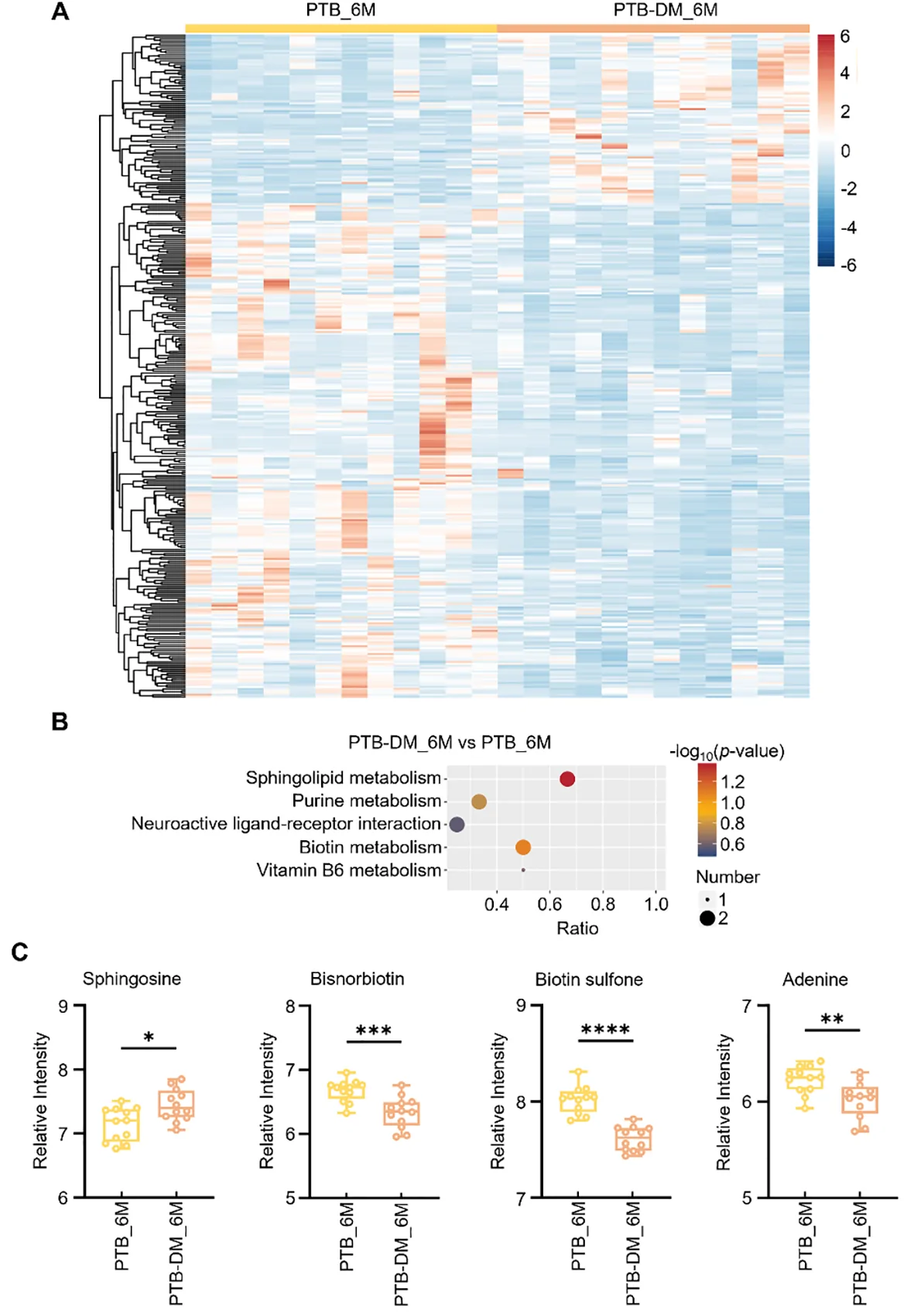
Persistent post-treatment metabolic abnormalities associated with comorbid T2DM. **(A)** Heatmap showing the relative abundance of differentially expressed metabolites between the PTB-DM_6M and PTB_6M groups. **(B)** Bubble plot presenting the top five KEGG pathways significantly enriched among differentially expressed metabolites. **(C)** Relative abundance of representative differentially expressed metabolites from different enriched pathways. Data are presented as mean ± SD; *p < 0.05, **p < 0.01, ***p < 0.001, ****p < 0.0001. PTB, pulmonary tuberculosis; DM, diabetes mellitus; KEGG, Kyoto Encyclopedia of Genes and Genomes; SD, standard deviation.

We investigated the distinct post-treatment metabolic features associated with comorbid T2DM by comparing the PTB_6M and PTB-DM_6M groups (**Figure 5A**). In contrast to the treatment-associated pattern observed in PTB alone, patients with PTB-DM retained a distinct post-treatment metabolic profile characterized by abnormalities in sphingolipid, purine, biotin, and vitamin B6 metabolism (**Figure 5B**; **Supplementary Table 12**). Notably, sphingosine level was increased and bisnorbiotin, biotin sulfone, and adenine levels were decreased in the PTB-DM_6M group compared with the PTB_6M group (**Figure 5C**). Collectively, these findings indicate that post-treatment metabolic differences between PTB-DM and PTB_6M were mainly associated with sphingolipid, purine, and biotin metabolism. Sphingosine, bisnorbiotin, biotin sulfone, and adenine were representative metabolites contributing to these pathway alterations.

### 3.7 Integrative analysis of shared and persistent metabolic alterations

**Figure 6:**
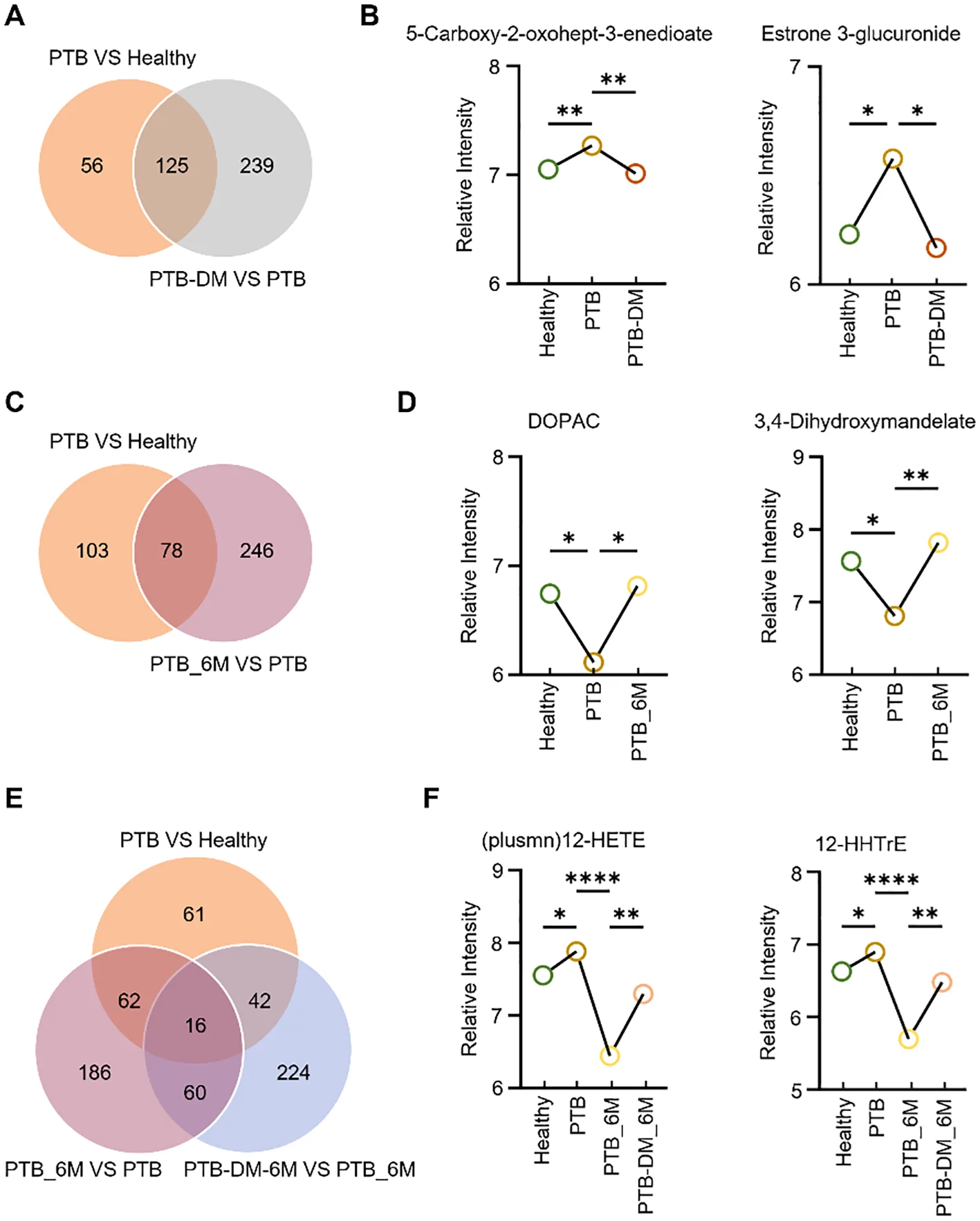
Integrative analysis of shared and persistent metabolic alterations. **(A)** Venn diagram showing overlapping differentially expressed metabolites between the PTB and Healthy groups and between the PTB-DM and PTB groups. **(B)** Relative abundance of representative shared differentially expressed metabolites. **(C)** Venn diagram showing shared differentially expressed metabolites between the PTB and Healthy groups and between the PTB_6M and PTB groups. **(D)** Relative abundance of representative shared differentially expressed metabolites. **(E)** Venn diagram showing shared differentially expressed metabolites across three comparisons (PTB vs Healthy, PTB_6M vs PTB, and PTB-DM_6M vs PTB_6M). **(F)** Relative abundance of representative shared differentially expressed metabolites. Data are expressed as mean ± SD; *p < 0.05, **p < 0.01, ****p < 0.0001. PTB, pulmonary tuberculosis; DM, diabetes mellitus; SD, standard deviation.

Intersection analyses were performed across different group comparisons to identify shared disease- and treatment-associated metabolic alterations. In total, 125 shared DEMs were identified between the PTB and Healthy groups and between the PTB-DM and PTB groups (**Figure 6A**; **Supplementary Table 13**). These shared metabolites were primarily enriched in tyrosine metabolism and steroid hormone biosynthesis (**Supplementary Table 14**). Notably, 5-carboxy-2-oxohept-3-enedioate and estrone 3-glucuronide levels were elevated in patients with PTB, whereas they were decreased in patients with PTB-DM (**Figure 6B**). Similarly, 78 metabolites were shared between the PTB and Healthy groups and between the PTB_6M and PTB groups (**Figure 6C**; **Supplementary Table 15**); these metabolites were predominantly related to the tyrosine metabolic pathway (**Supplementary Table 16**). The levels of DOPAC and 3,4-DHM were lower in patients with PTB and higher in the PTB_6M group (**Figure 6D**). Intersection analyses comparing the PTB vs. Healthy, PTB_6M vs. PTB, and PTB-DM_6M vs. PTB_6M groups identified 16xcommon metabolites (**Figure 6E**; **Supplementary Table 17**) that were enriched in arachidonic acid metabolism and related pathways (**Supplementary Table 18**). Among them, (±)12-.hydroxyeicosatetraenoic acid (12-HETE) and 12-hydroxyheptadecatrienoic acid (12-HHTrE) levels were elevated in the PTB group and, despite declining after treatment, remained persistently high in the PTB-DM_6M group (**Figure 6F**). Collectively, these findings indicate that some metabolites are consistently associated with PTB, PTB-DM, and post-treatment states. 12-HETE and 12-HHTrE showed persistent alterations across disease and post-treatment comparisons, suggesting that these metabolites warrant further validation as candidate indicators of PTB-associated metabolic alterations.

**Figure 7:**
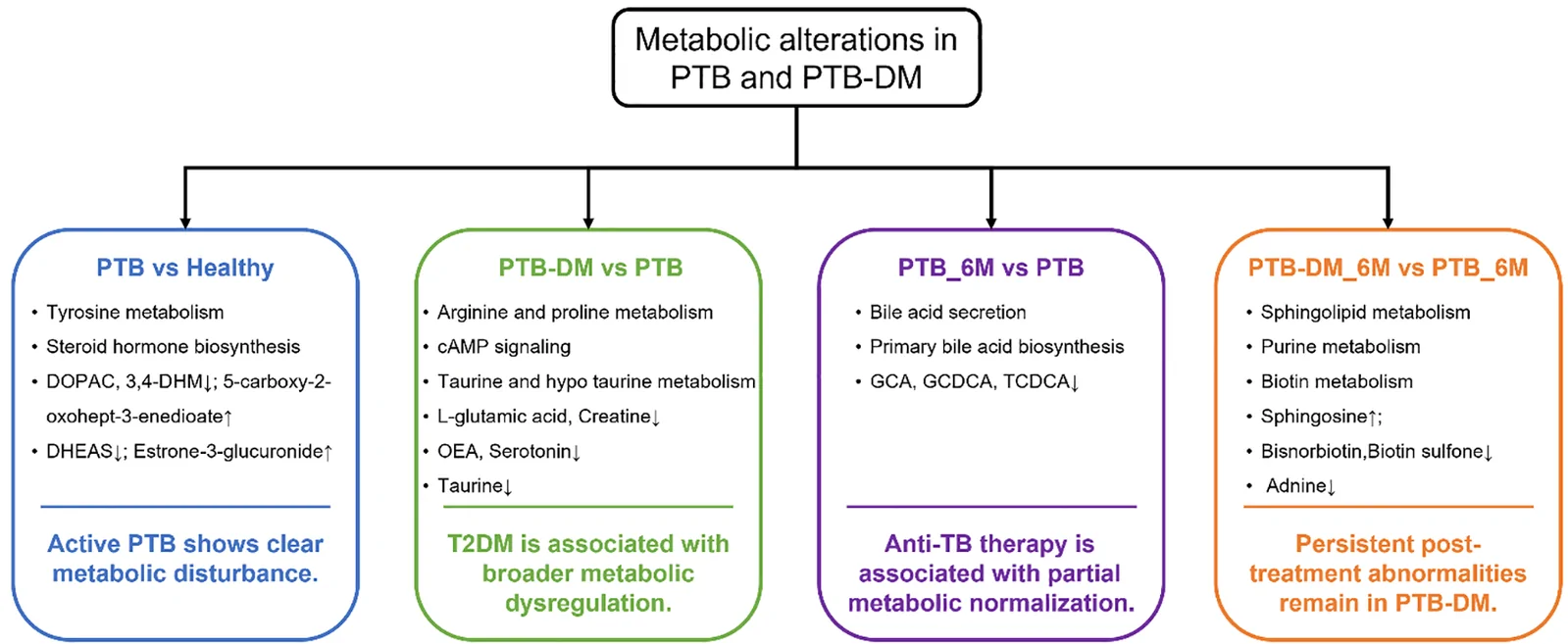
Summary of the major metabolic alterations. Compared with healthy controls, active PTB was associated with alterations in tyrosine metabolism and steroid hormone biosynthesis. Compared with PTB alone, PTB-DM was associated with broader metabolic perturbations involving arginine and proline metabolism, cAMP signaling, taurine and hypotaurine metabolism, and related amino acid and energy-associated metabolites. In patients with PTB, the comparison between the active-disease and independent 6-month post-treatment groups identified prominent treatment-associated changes in bile acid secretion and primary bile acid biosynthesis. After treatment, patients with PTB-DM retained distinct abnormalities in sphingolipid, purine, biotin, and vitamin B6 metabolism compared with patients with PTB alone. PTB, pulmonary tuberculosis; T2DM, type 2 diabetes mellitus; PTB-DM, PTB patients with comorbid T2DM; GCA, glycocholic acid; GCDCA, glycochenodeoxycholic acid; TCDCA, taurochenodeoxycholic acid; DOPAC, 3,4-dihydroxyphenylacetic acid; DHEAS, dehydroepiandrosterone sulfate; OEA, oleoylethanolamide.

Collectively, these analyses delineate a hierarchical pattern of metabolic alterations across disease status, diabetes comorbidity, and treatment stage (**Figure 7**). Active PTB was primarily associated with alterations in tyrosine metabolism and steroid hormone biosynthesis, whereas concomitant T2DM was associated with broader perturbations involving amino acid metabolism, cAMP signaling, and taurine-related metabolism. In patients with PTB, the comparison between the active-disease and independent 6-month post-treatment groups revealed prominent differences in bile acid-related metabolism. Moreover, patients with PTB-DM exhibited a distinct post-treatment metabolic profile characterized by abnormalities in sphingolipid, purine, biotin, and related metabolic pathways compared with patients with PTB alone.

## 4 Discussion

In this study, untargeted plasma metabolomics was applied to characterize metabolic alterations associated with active PTB, comorbid T2DM, and post-treatment states after standard anti-TB therapy. We found that active PTB exhibited distinct metabolic alterations, particularly involving tyrosine metabolism and steroid hormone biosynthesis. Compared with PTB, PTB-DM showed additional metabolic perturbations mainly involving arginine and proline metabolism, steroid hormone biosynthesis, cAMP signaling, and taurine and hypotaurine metabolism. In addition, comparison between PTB and PTB_6M groups revealed post-treatment metabolic differences mainly associated with bile acid metabolism. Furthermore, PTB-DM_6M remained associated with distinct post-treatment metabolic differences involving sphingolipid, purine, and biotin metabolism compared with PTB_6M. Intersection analyses further identified shared metabolic alterations across disease and post-treatment states, including persistent changes in lipid mediator-related metabolites such as 12-HETE and 12-HHTrE, highlighting their potential relevance for further investigation as biomarkers of PTB-associated metabolic alterations. Collectively, these findings suggest that T2DM is associated with broader metabolic disturbances during active PTB and persistent metabolic differences after anti-TB therapy, although the underlying biological mechanisms require further investigation.

Compared to healthy controls, patients with PTB exhibited significant alterations in tyrosine metabolism and steroid hormone biosynthesis. The downregulation of DOPAC and 3,4-DHM, together with the increase in 5-carboxy-2-oxohept-3-enedioate levels, may reflect altered catecholamine-related tyrosine metabolism. DOPAC, a dopamine catabolite, mitigates mitochondrial oxidative injury (Catalan et al., 2020; Tang et al., 2016), whereas 3,4-DHM is a norepinephrine metabolite (Yuan et al., 2020). Elevated urinary norepinephrine levels have been observed in patients with PTB (Das et al., 2015), and dopamine β-hydroxylase deficiency increases the susceptibility to PTB infection (Alaniz et al., 1999). The elevated 5-carboxy-2-oxohept-3-enedioate level suggests enhanced tricarboxylic acid cycle flux, thereby facilitating energy production and redox balance under inflammatory stress (Collins et al., 2021; Gao et al., 2023). In the present study, DHEAS levels were decreased in the steroid hormone biosynthesis pathway, whereas estrone 3-glucuronide levels were increased. Estrone 3-glucuronide, an estrogen conjugate, exerts anti-inflammatory effects (Li et al., 2022; Lindsey, 2014). In contrast, DHEAS regulates immune function and antimicrobial defense (Wu et al., 2014). The emergence of a “high-cortisol–low-DHEAS” hormonal pattern has been associated with T-cell dysfunction and chronic inflammation (Bozza et al., 2007; Tsegaye et al., 2022). After 6 months of standard anti-TB therapy (2HRZE/4HR), patients with PTB exhibited extensive metabolic remodeling, primarily involving bile secretion, primary bile acid biosynthesis, and cholesterol metabolism. This remodeling was accompanied by significant reductions in GCA, GCDCA, and TCDCA levels compared with the pre-treatment levels. Bile acids act as signaling molecules by binding to FXR and TGR5 receptors, thereby regulating energy metabolism and inflammation (Pols et al., 2011a, Pols et al., 2011b). Therefore, reduced bile acid levels after treatment may reflect a shift away from an inflammatory, high-flux metabolic state and may be related to reduced oxidative stress and lipid metabolic remodeling (Li et al., 2024; Zhang et al., 2006).

Compared with PTB patients, PTB-DM patients exhibited additional metabolic alterations, mainly involving arginine and proline metabolism, steroid hormone biosynthesis, cAMP signaling, and taurine and hypotaurine metabolism. The reduction in L-glutamic acid levels may reflect altered glutathione-related metabolism, consistent with impaired glutathione metabolism in T2DM (Lutchmansingh et al., 2018). The reduction in creatine levels may suggest alterations in the creatine–phosphocreatine energy shuttle, paralleling diabetes-associated creatine depletion (Nie et al., 2018). The decrease in OEA levels reflects disrupted lipid signaling and may be linked to enhanced NLRP3 inflammasome activation and persistent inflammation under insulin-resistant conditions (Devi et al., 2019; Hu et al., 2020). The decline in serotonin and taurine levels may reflect changes in neuroactive metabolite-related signaling and antioxidant-related metabolism (Wu et al., 2019, Wu et al., 2022; Zhou et al., 2019). Notably, accumulating evidence indicates that successful microbiological cure of tuberculosis does not necessarily equate to complete restoration of pulmonary homeostasis. Even after standard anti-tuberculosis therapy, a substantial proportion of patients develop persistent structural and functional sequelae, including chronic airflow limitation and post-tuberculosis lung disease (Gai et al., 2024). PTB-DM_6M patients exhibit abnormalities in sphingolipid, purine and biotin metabolism than PTB_6M patients. Sphingolipids and their metabolites play a crucial role in regulating fundamental cellular processes, including proliferation, apoptosis, survival and migration (Mao, 2025). Increased sphingosine levels indicate defective membrane-associated signaling and chronic inflammation (Hannun and Obeid, 2018). Decreased bisnorbiotin and biotin sulfone levels suggest attenuated coenzyme-dependent carboxylation and impaired fatty-acid oxidation (Jitrapakdee and Wallace, 2003; Leon-Del-Rio, 2019); and reduced adenine levels indicate restricted nucleotide turnover (Tran et al., 2024). Collectively, these data indicate that the post-treatment metabolic abnormalities observed in patients with PTB-DM are consistent with previously reported.

From a clinical perspective, persistent post-treatment metabolic abnormalities may indicate that microbiological cure does not necessarily coincide with complete normalization of host metabolic homeostasis. Such incomplete metabolic normalization may represent a metabolic correlate of persistent host dysfunction after anti-TB therapy, potentially related to residual inflammation or impaired tissue repair (Andersen, 2022; Velur et al., 2025). These persistent host alterations may contribute to post-tuberculosis lung disease (PTLD), including pulmonary fibrosis, bronchiectasis, and cavitation (Yadav and Rawal, 2024). However, the present study did not directly assess lung function recovery, PTLD, or other long-term clinical outcomes.

Intersection analyses identified shared differential metabolites across multiple comparisons, providing additional insights into metabolic alterations associated with PTB-DM and post-treatment states. The levels of 5-carboxy-2-oxohept-3-enedioate and estrone 3-glucuronide were elevated in patients with PTB but decreased in those with PTB-DM. These findings suggest that tyrosine degradation-related and steroid hormone-associated metabolic pathways may be differentially regulated in PTB and PTB-DM. Previous studies have linked tyrosine metabolism and steroid hormone metabolism to metabolic adaptation and immune-endocrine regulation, suggesting that the altered patterns observed in PTB-DM may reflect differences in aromatic amino acid metabolism and hormone-related metabolic responses under diabetic conditions (Bala et al., 2021; Bukato et al., 2024; Chen et al., 2022; Tataru et al., 2025). In addition, DOPAC and 3,4-DHM levels were lower in active PTB and higher in the post-treatment PTB group. During active TB infection, persistent inflammatory and oxidative stress conditions may alter amino acid metabolic pathways, potentially affecting the availability of tyrosine for catecholamine-related metabolism and contributing to reduced downstream metabolites such as DOPAC and 3,4-DHM (Cappelletti et al., 2023; Hufner et al., 2023). After anti-TB therapy, changes in the inflammatory environment may be associated with altered tyrosine-related catecholamine metabolic profiles (Gietl et al., 2024). However, whether these metabolic differences reflect functional changes during host recovery requires further investigation. Notably, 12-HETE and 12-HHTrE levels were elevated in patients with PTB and decreased after treatment, whereas they remained high in patients with PTB-DM after treatment. Both metabolites are derived from arachidonic acid oxidation pathways and represent important components of lipid mediator networks involved in inflammatory signaling. The sustained alterations of 12-HETE and 12-HHTrE may suggest persistent differences in lipid mediator-associated metabolism and inflammatory lipid signaling in patients with PTB-DM after treatment (Ding et al., 2024; Jontvedt Jorgensen et al., 2021; Poznyak et al., 2020). Therefore, persistent alterations in these metabolites may indicate sustained differences in lipid mediator-related metabolism associated with PTB-DM after anti-TB therapy, although inflammatory resolution was not directly assessed in this study.

This study had certain limitations. The sample size was modest and derived from a single center, which may limit the generalizability of the findings. Although the between-group differences in age and sex distribution did not reach statistical significance, residual confounding by these demographic factors cannot be excluded. Age and sex may influence circulating steroid hormone, lipid, amino acid, and energy-related metabolites. Variations in smoking history, glycemic control, medication use, dietary intake, nutritional status, hormonal status, body composition, and coexisting metabolic conditions may have influenced the observed metabolic patterns. Although untargeted metabolomic analysis provides broad biochemical coverage, targeted quantification is required to validate key metabolites and effect sizes. The baseline and 6-month post-treatment groups were analyzed as independent groups rather than paired longitudinal samples, limiting our ability to define within-subject metabolic trajectories across the treatment course. Although candidate differential metabolites were screened using a combined VIP, FC, and p-value threshold, several associations did not remain significant after FDR correction. These findings should therefore be considered exploratory and require validation in larger cohorts and by targeted quantitative metabolomic assays. Because this study was based on plasma metabolomics, immune recovery, antimicrobial defense, endocrine stress adaptation, inflammatory resolution, neuroimmune regulation, and tissue repair were not directly assessed. Accordingly, this untargeted metabolomic analysis should be regarded as exploratory and hypothesis-generating.

## 5 Conclusions

In summary, this study suggests that PTB is associated with distinct metabolic alterations across active and post-treatment states, whereas comorbid diabetes is associated with broader metabolic dysregulation and persistent post-treatment metabolic differences. These alterations may be related to incomplete re-establishment of metabolic homeostasis following anti-TB therapy, although immune homeostasis was not directly assessed in this study. Nonetheless, future multicenter, longitudinal studies integrating targeted metabolomics, immune profiling, and clinical outcome assessment are warranted to confirm and extend these findings.

## Data Availability

The raw metabolomics data generated in this study have been deposited in the China National Gene Bank Sequence Archive (CNSA: CNP0008986; https://db.cngb.org). Additional data supporting the findings of this study are available from the corresponding author upon reasonable request.
